# Partial waiver of consent to overcome translational science barriers in neonatal clinical research

**DOI:** 10.1017/cts.2025.10122

**Published:** 2025-08-27

**Authors:** Megha Sharma, Sarah E. Diamond, Jenna Chancellor, Simon Chung, Andrew W. Brown, D. Micah Hester, Laura P. James, Mario Schootman, Peter M. Mourani, Sherry E. Courtney

**Affiliations:** 1 Division of Neonatology, Department of Pediatrics, University of Arkansas for Medical Sciences, Little Rock, AR, USA; 2 Arkansas Children’s Research Institute, Little Rock, AR, USA; 3 Department of Biostatistics, University of Arkansas for Medical Sciences, Little Rock, AR, USA; 4 Department of Medical Humanities and Bioethics, University of Arkansas for Medical Sciences, Little Rock, AR, USA; 5 Division of Emergency Medicine, Department of Pediatrics, University of Arkansas for Medical Sciences, Little Rock, AR, USA; 6 Translational Research Institute, University of Arkansas for Medical Sciences, Little Rock, AR, USA; 7 Institute of Community Health and Innovation, Springdale, AR, USA; 8 Division of Critical Care, Department of Pediatrics, University of Arkansas for Medical Sciences, Little Rock, AR, USA

**Keywords:** Neonates, clinical trials, consent, equity, enrollment

## Abstract

Prospective consent in neonatal research poses significant challenges, particularly during urgent, time-sensitive clinical windows of study enrollment. This is especially true at referral centers for large geographic regions. A partial waiver of consent offers a potential translational science approach to enhance access to research participation in critically ill neonates. We compared enrollment rates in a study evaluating pulse oximetry accuracy across neonates with varying skin pigmentation before and after implementing a partial waiver of consent. Overall enrollment increased significantly without creating a racial disparity in enrollment, thereby improving generalizability and efficiency in neonatal clinical research.

## Introduction

Informed consent in human subjects research is a process by which “a subject voluntarily confirms his or her willingness to participate in a particular study, after having been informed of all aspects of the study that are relevant to the subject’s decision to participate” [1]. In neonatal intensive care units (NICUs), consent must be obtained from a parent or legal guardian, as the research subjects are typically critically ill neonates. Caregivers, particularly postpartum mothers, often face emotional distress, fear, and unfamiliarity with the NICU environment as well as logistical barriers such as having to remain hospitalized at the birth hospital while their neonate is transferred to a tertiary care center, making meaningful consent discussions difficult. Antenatal consent can alleviate some of these barriers if the study conditions or eligibility can be anticipated [2]. However, when critical illness of a neonate is unpredictable or when research occurs at tertiary care centers separate from delivery units, timely consent is difficult, especially for conditions with a time-sensitive enrollment window. A substantial proportion of clinical trials experience delays, exceed budget projections, or are terminated early due to inadequate participant recruitment, thereby limiting scientific validity and failing to generate knowledge generalizable to specific populations [3,4]. In neonatal/critical care clinical research, low enrollment and insufficient racial and ethnic diversity remain persistent challenges, undermining the generalizability of study findings [5–7]. Despite ongoing efforts to improve research enrollment through methods such as remote or telephone consent, a diverse recruitment workforce, and prenatal consent, substantial barriers to participation persist, particularly among racial and ethnic minority populations [8,9]. Translational science aims to address such roadblocks (i.e., ineffective recruitment) in the research process, catalyzing success and overcoming common barriers in a disease-agnostic approach [10,11].

In a recent prospective study evaluating the accuracy of pulse oximetry in neonates of varying skin pigmentation by comparing to the gold standard arterial blood gas oximetry results, we first used a traditional prospective consent approach. This was a minimal risk study with a time-sensitive window of eligibility and involved recruiting the most critically ill neonates from the ICU soon after birth. However, our prospective consent approach proved scientifically limiting due to: (1) narrow, limited window of eligibility for when arterial lines are present (typically within first 3–4 postnatal days), (2) overwhelmed parents/guardians suffering intense emotional distress, (3) disproportionately greater challenges in reaching out to and enrolling mothers facing systemic barriers (socioeconomically disadvantaged, inability to travel to the hospital, phone/internet connectivity), and (4) inefficient use of research resources and participant exclusion by the time consent was obtained, due to removal of the arterial line. With this approach, enrollment rates remained low: only 50%–60% of eligible patients could be approached, and just 33% of the eligible population was ultimately enrolled in this minimal-risk study, reflecting significant missed opportunities for participation. Data collection could not begin until consent was signed. Thus, the time to complete the process of informed consent delayed data collection in the consented infants and caused many others to become ineligible before their data could be collected. Our team then worked with our institutional review board (IRB) to effectively and ethically use altered consent practices with the goal of enhancing participant recruitment. Pulse oximeters in current use were inadequately calibrated and validated in dark skin pigmented individuals, and based on some recent studies, may negatively impact these patients by providing inaccurate readings [12–15]. Therefore, any alteration in consent process would have to be designed with careful consideration of not perpetuating the same representational gaps seen in earlier pulse oximetry research, namely, the inadequate inclusion of participants with darker skin tones.

Under certain circumstances, informed consent can be waived, and “research with altered consent process” is possible, allowing the enrollment of participants meeting strict regulatory conditions. Under Title 45 of the Code of Federal Regulations 45 CFR 46.11616, in order for research to be conducted with a waived or altered consent, four conditions must be met: “(1) research involves no more than minimal risk to subjects; (2) the waiver or alteration will not adversely affect rights and welfare of subjects; (3) research could not practicably be carried out without the waiver or alteration; and (4) whenever appropriate, subjects will be provided with additional pertinent information after participation” [16]. In addition, the study protocol must uphold the principle of beneficence by “minimizing non-therapeutic procedures and ensuring their necessity is justified in relation to knowledge gained.” While it is ideal to approach parents only when they are physically and emotionally ready to engage in decision-making, this well-intentioned delay can unintentionally hinder participation and contribute to missed research opportunities, especially in studies involving minimal risk and time-critical enrollment and/or data collection windows. In response, we explored using a partial waiver of consent to address a long-standing critical barrier in NICU-focused time-sensitive research.

## Methods

The primary study, the AR-Neo-PODS study (Arkansas- Neonatal – Pulse Oximetry Disparity Study), collects vital signs (pulse oximetry) and blood gas values (arterial oxygen saturation as the gold standard) to assess the extent of pulse oximetry inaccuracy among infants of varying skin pigmentation. The “research” addition to this study involves objective skin pigment assessment using a painless, non-invasive colorimeter, SkinColorCatch (Delfin, Miami, FL) which involves a brief one-second touch to the neonate’s skin at the site of pulse oximeter sensor (foot or hand). Eligible patients were those that were >25 weeks postmenstrual age at birth and had both arterial lines and blood gases ordered, so we could compare pulse oximeter readings against the blood gas results. The study was conducted at Arkansas Children’s Hospital (ACH), the only level 4 NICU in the state of Arkansas. As the state’s only designated tertiary care center for critically ill neonates, ACH receives transfers from all birthing hospitals in Arkansas, the majority of which are located at considerable distances.

Our standard approach is to call or meet the mother in person and ask which caregiver(s) she would like included in the consent discussion. Consent is ultimately obtained from the mother or father (for married couples). Skin pigment assessments and data collection would commence only after consent was obtained. After an initial phase of 10 months of this traditional prospective consent process, our IRB granted approval for a partial waiver of consent (sometimes referred to as “delayed consent”) to collect minimal risk data (vital signs, blood gas, demographics), and skin pigmentation (melanin estimates) using the colorimeter. Where possible, parents were approached for consent before data were collected. In cases in which parents or guardians could not be reached in person or by phone at the time of eligibility, contact was attempted as soon as possible and no later than hospital discharge. In these cases, study data could be collected in a timely fashion from when eligibility was identified and seek consent after data collection had begun. Only data from neonates whose parents consented were retained. Data were destroyed for those whose parents declined or could not be reached. In our study, all consent was provided by biological parents.

We compared enrollment rates, defined as the number of neonates consented divided by those approached, before (Sept 2023–June 2024) and after (July 2024–Feb 2025) the partial waiver (PW) implementation. We also compared enrollment results by race-ethnicity (EHR-reported maternal race) since it was important to monitor our recruitment efforts to make sure we recruited a wide spectrum of skin pigmentation. In the pre-PW phase, “approached” infants were those for whom the research team initiated prospective consent process (i.e., initiating contact) with a parent or legal guardian. In the PW phase, “approached” infants implied those who underwent study-related procedures and data collection. Enrolled infants were defined as those for whom consent (prospective or delayed, depending on the phase) was successfully obtained. Our primary outcome was enrollment rate (enrolled ÷ approached × 100), which was compared across both phases and stratified by race using a two-sided, two-sample proportion test. Secondary analyses modeled enrollment rate as a function of race, phase, and their interaction using logistic regression. A statistically significant interaction would be evidence of changes in the enrollment rate that varied by race. We included only NHW and NHB infants because these groups had large enough samples to compare pre- and post-changes within each race. Multiple comparison correction is omitted given the targeted and limited number of tests, and all comparisons of interest are reported for transparency. Analyses were performed in R version 4.4.3, with p-values <0.05 considered statistically significant.

## Results

**Figure 1.**
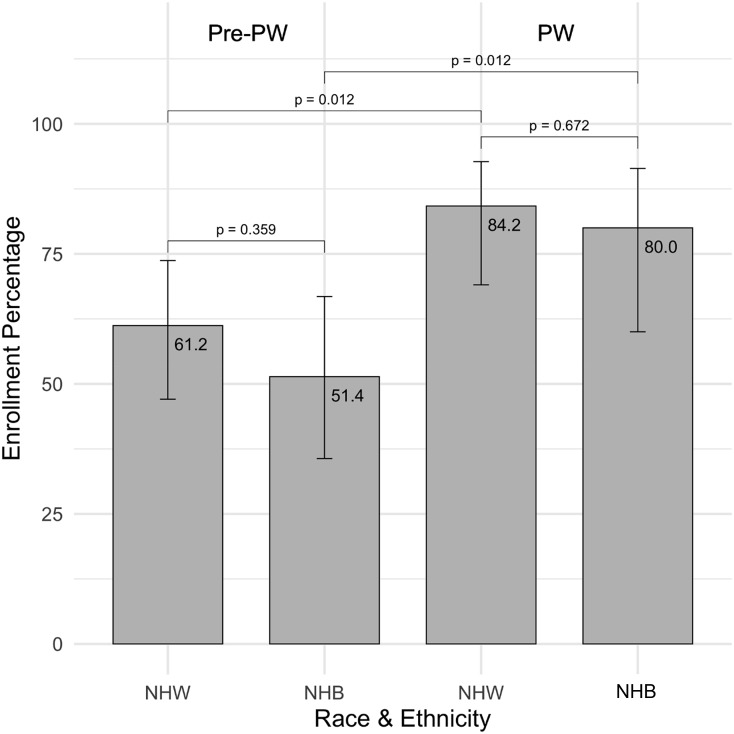
Implementing partial waiver of consent was associated with improved enrollment among neonates born to non-Hispanic White and non-Hispanic Black mothers. The figure shows estimated enrollment percentages by race and phase (Pre–Partial waiver [Pre-PW] vs. Partial waiver [PW]), derived from a logistic regression model with an interaction term. Error bars represent 95% confidence intervals. P-values reflect pairwise comparisons of model-estimated proportions. Neither the between-race comparisons within each phase nor the interaction term (*p* = 0.886) was statistically significant.

The number of infants eligible with an arterial line for blood gas assessment was 181 in the pre-PW (10 months) and 94 in the PW (8 months) cohorts (Table 1). The number of infants approached for study assessments increased from 55% (100/181) in the pre-PW to 81.9% (77/94) in the PW phase (*p* < 0.001). Implementing partial waiver of consent improved the overall enrollment rate across all races from 60.0% (60/100) to 80.5% (62/77) (*p* = 0.006). Enrollment rates increased from 61.2% (30/49) to 84.2% (32/38) among NHW infants (*p* = 0.035), and from 51.4% (19/37) to 80.0% (20/25) among NHB infants (*p* = 0.043; Table 1). As a secondary analysis, logistic regression including the race-phase interaction showed results consistent with the stratified proportion tests. Neither the between-race comparisons within each phase nor the interaction term was statistically significant (Figure 1). Informal caregiver feedback during consent discussions indicated that the majority of parents that declined participation did so because they felt overwhelmed and stressed by their infant’s critical condition. A smaller number expressed general mistrust or disinterest in participating in any form of research, or discomfort with researchers retaining their child’s health information. For parents who provided consent, the majority reported no hesitations, citing the absence of additional interventions or procedures involving their infant. During the PW phase, many of those who agreed to participate using “delayed consent” expressed appreciation for the timing of the consent process, noting that they felt less overwhelmed at that point.

**Table 1.** Enrollment rates of infants before and after implementing partial waiver of consent process in the AR-neo-PODS study

	Pre-Partial waiver of consent		Partial waiver of consent		
Race-Ethnicity	Eligible	% Enrollment Rate ^#^ (N of Consented/Approached^)	Eligible	% Enrollment Rate ^#^ (N of Consented/Approached^^^)	*P-value*
Non-Hispanic White	89	61.2 (30/49)	44	84.2 (32/38)	0.035
Non-Hispanic Black	50	51.4 (19/37)	27	80.0 (20/25)	0.043
Hispanic*	29	90.0 (9/10)	14	80.0 (4/5)	–
Other*	13	50.0 (2/4)	9	66.6 (6/9)	–
Total	181	60.0 (60/100)	94	80.5 (62/77)	0.006

# Statistical analysis was conducted for enrollment rate (consented “Yes” divided by those approached) in pre-partial waiver (Pre-PW) and partial waiver (PW) phases.

* Represents data not statistically analyzed due to low numbers of consented and approached. Race-Ethnicity: refers to maternal race-ethnicity recorded in the Electronic Health Record per U.S. Office of Management and Budget (OMB) standards. Reference: Office of Management and Budget. (2024). *Statistical Policy Directive No. 15: Standards for Maintaining, Collecting, and Presenting Federal Data on Race and Ethnicity.*

^ Approach rate significantly increased from pre-PW 55.2% to PW 81.9% (two-sample proportion test, *p* < 0.001) in the post-PW compared to pre-PW phase.

Reasons for low approach rates in pre-PW phase:.

(1) Inability to reach the parents.

(2) Loss of eligibility by the time parental contact was made.

(3) Significant clinical instability.

(4) Language barriers.

(5) Teenage parents.

(6) Social barriers, such as custody issues or protective holds.

## Discussion

The overall purpose of the present study is to investigate the presence and extent of pulse oximetry inaccuracy relative to skin pigmentation. A diverse and adequate representation of neonates from different races and skin tones was essential in addressing the study’s objective. However, recruitment challenges resulted in missed opportunities across all participant groups, reflecting the complexity of obtaining prospective parental consent in the acute neonatal time-critical period. Implementing a partial waiver of consent helped reduce these major barriers to the participation of infants overall, without creating an enrollment disparity between NHW and NHB neonates. The enrollment rates increased without requiring additional effort or financial resources for study execution while also easing potential burdens on the parents of the neonates.

Observations noted during the pre-PW recruitment process pointed to several practical challenges that contributed to missed enrollment opportunities. While these were not formally evaluated, they informed our decision to adopt a partial waiver approach to reduce time-sensitive barriers. A substantial amount of time, effort, and resources were devoted to approaching postpartum mothers/parents for consent during the pre-PW phase. When successfully approached, the consent discussions were relatively uncomplicated given the minimal risk, non-invasive nature of our study. However, by the time consent was obtained, the narrow window for accessing the infant’s arterial line and collecting data for the study, typically limited to the first few days after birth or periods of acute critical illness, was often missed. These clinical windows often coincided with time periods of “situational incapacity” when families were in acute emotional distress [17]. Much of neonatology research occurs in Level 4 ICUs (tertiary care centers), where postpartum mothers/parents are frequently located at a separate birthing hospital posing additional logistical barriers for time-sensitive research. The “alteration of consent” gave families more time to meet with study team members in an unpressured manner facilitating an enhanced exchange of information. A recent systematic review of neonatal clinical trials registered in the Cochrane Central database revealed that less than half (∼44%) of eligible infants are enrolled in neonatal clinical trials [6], and there is substantial under-representation of neonates born to Black, Asian, Indigenous, and Hispanic parents compared to their proportion within the U.S. ICU population [5]. The most commonly cited reasons for declining enrollment were parental refusal followed by parents not being accessible for approach [7]. These findings underscore the need for interventions to improve the timing and communication of consent processes, as well as a deeper examination of why some families are not accessible for approach, within the specific context and clinical environment of a particular study [6].

Alterations of consent process (such as a waiver) are sometimes sought in emergency and critical care studies needing urgent interventions [18]. In the Time to Reduce Mortality in End-Stage Renal Disease (TiME) study, an opt-out approach was used whereby potential participants were included in the research process unless they decided not to participate [19]. The Oxy-PICU study compared two oxygen saturation targets in critically ill children (mean age: 2.5 y) with deferred consent after randomization. The study reported a “declined consent” rate under 10%; 91% of surveyed parents reported satisfaction with the consent process [20]. Deferred consent was used in a UK-based study that randomized critically ill children in pediatric ICUs to evaluate different central venous catheters within an emergent time-critical window. Detailed mixed-methods evaluation of views and acceptability of deferred consent process in this study that enrolled children beyond the neonatal period (majority >3 months of age) found that a small percentage of parents initially reported feeling “in dismay” or “surprised” by enrollment through deferred consent. However, none reported “dissatisfaction with the consent process” and all parents reported that their initial concerns were short-lived and mitigated by the minimal risk nature and time-sensitive nature of the study [21]. In that same study, parents further reported that timing, clinical context, and the quality of communication were the most important factors influencing their willingness to participate. The consent strategy in our study echoed similar considerations, with an approach structured to respect parental needs and support ethical study participation.

Our study has several strengths. Our study demonstrated that a partial consent waiver allowed us to overcome a well-documented translational barrier in neonatal research, i.e., the difficulty of obtaining timely consent from post-partum mothers within narrow eligibility and data collection windows. Moreover, the nature of many tertiary neonatal care centers is that post-partum mothers frequently remain in smaller, referring hospitals that span a driving distance of several hours from the tertiary/quaternary NICU. Our study extends the use of partial waiver methods to its application and effective implementation in a high-acuity, tertiary care, state-wide referral-based neonatal center of a rural state. This approach enabled us to meet the study’s population-based recruitment goals and reduce missed research opportunities. In this regard, our study illustrates application of four of the seven foundational principles of translational science defined by the National Center for Advancing Translational Sciences: (1) addressing unmet needs, (2) creating generalizable solutions to persistent challenges, (3) promoting innovation in research practices, and (4) accelerating the translational process [10]. These principles were integrated with core bioethical considerations surrounding informed consent to guide our approach.

Our study is not without limitations. Our team did not engage a former NICU parent or community advisory board to understand which recruitment strategies might be considered acceptable before implementing this change [17]. We did not formally include or analyze the perspectives of parents and key stakeholders such as study investigators, research coordinators, and clinical teams in the form of qualitative interviews, surveys or focus groups. We did not systematically measure socioeconomic influences that affected the enrollment process. These things are important future work that must be done for many trials, but cannot be routinely done due to limitations in time and resources. However, we did seek informal feedback on why parents or guardians declined to have data included which informed the “alteration of consent” process. We limited our comparative analysis to enrollment rates between NHB and NHW groups due to limited sample size of other races. Given the pre- and post-nature of our study, we cannot be certain if other factors contributed to this increase in enrollment, though the composition of the research team (experience, racial demographics) was similar in both phases. Finally, the rationale for and potential generalizability of implementing a similar approach may vary depending on the risk category and type of study (observational vs. interventional), as well as the specific regulations and institutional policies applicable within different states, countries, or research domains.

This report highlights a promising, pragmatic, and ethical strategy that can serve as a catalyst for improving the pace and equity of participant enrollment in NICU time-sensitive research. Beyond the NICU, this approach may be adaptable to other pediatric or acute care research settings, particularly within statewide referral-based systems in under-resourced or rural states facing similar challenges.
